# FAM13A promotes proliferation of bovine preadipocytes by targeting Hypoxia-Inducible factor-1 signaling pathway

**DOI:** 10.1080/21623945.2021.1986327

**Published:** 2021-10-21

**Authors:** Chengcheng Liang, Guohua Wang, Sayed Haidar Abbas Raza, Xiaoyu Wang, Bingzhi Li, Wenzhen Zhang, Linsen Zan

**Affiliations:** a College of Animal Science and Technology, Northwest A&f University, Yangling, P.R. China; b National Beef Cattle Improvement Center, Northwest A&F University, Yangling, P.R. China

**Keywords:** FAM13A, hypoxia, proliferation, hif-1 pathway

## Abstract

The family with sequence similarity 13 member A (FAM13A) gene has been discovered in recent years and is related to metabolism. In this study, the function of FAM13A in precursor adipocyte proliferation in Qinchuan cattle was investigated using fluorescence quantitative polymerase chain reaction (PCR), western blotting, 5-ethynyl-2′-deoxyuridine staining, and other tests. FAM13A promoted precursor adipocyte proliferation. To determine the pathway FAM13A was involved in, transcriptome sequencing, fluorescence quantitative PCR, western blotting, and other tests were used, which identified the hypoxia inducible factor-1 (HIF-1) signalling pathway. Finally, cobalt chloride, a chemical mimic of hypoxia, was used to treat precursor adipocytes. mRNA and protein levels of FAM13A were significantly increased after hypoxia. Thus, FAM13A promoted bovine precursor adipocyte proliferation by inhibiting the HIF-1 signalling pathway, whereas chemically induced hypoxia negatively regulated FAM13A expression, regulating cell proliferation.

## 1. Introduction

Beef has a high level of protein, minerals, and vitamins [1–3]. Beef that is rich in fat increases the added value; thus, the study of the molecular mechanism of intramuscular fat deposition is important to improve beef quality and molecular breeding of beef cattle [4–7]. Adipose tissue develops from adipocytes through cell differentiation and cell proliferation [8–11]. During precursor adipocyte development, cell proliferation is strictly regulated by the cell cycle, which is divided into the interphase and division phases [12]. A variety of intracellular factors and proteins have a regulatory role during cytokinesis, providing precise regulation of cell proliferation; these include the cell cycle-dependent kinases (CDK), the cyclins, and the cell cycle inhibitor protein [13].

The family with sequence similarity 13 member A (FAM13A) gene exhibits a high degree of sequence conservation [14]. Currently, chronic obstructive pulmonary disease (COPD) is highly studied in human medicine [15,16]. In 2017, FAM13A was investigated for its correlation with susceptibility to COPD [17]. In recent years, an increasing number of studies have associated FAM13A with metabolism; for example, FAM13A enhances insulin sensitivity in mice by maintaining homoeostasis of the body system by regulating insulin signalling in adipocytes [18]. In 2020, studies on FAM13A in mice have shown that knocking down the gene accelerates adipocyte differentiation and that knockout mice accelerate adipocyte differentiation and glucose uptake, while having better lipogenic differentiation potential [19–22]. Another study has shown that FAM13A regulates lipid metabolism, but deletion of this gene resists high-fat diet-induced fatty liver in mice [23]. Studies on FAM13A in cell proliferation are rarely reported, and most studies are focused on COPD and non-small cell lung cancer (NSCLC) [24,25]. Studies have reported that fam13a is related to the occurrence of NSCLC, and siRNA to FAM13A infected tumour cell promotion [26].

A certain oxygen concentration is essential for normal physiological activities of cells in aerobic organisms; during biological evolution, cells have evolved a regulatory mechanism for fluctuations in oxygen concentration within a certain range, known as hypoxic adaptation [27]. Hypoxia is involved in various cellular physiological processes, such as cell differentiation, normal brain functioning, and normal heart function [28,29]. Hypoxia-inducible factors (HIFs) are the main regulatory transcription factors involved in the maintenance of oxygen homoeostasis in aerobic organisms [30]. Cobalt chloride is a commonly used mimic of chemically induced hypoxia, and it stably induces HIF-1α expression [31]. The mechanism of action is the substitution of Co2+ with Fe2+ in prolyl hydroxylases (PHDs), key enzymes that correlate O2 concentration with HIF degradation under normal oxygen conditions [13]. The use of cobalt chloride to construct hypoxia models is widely used in scientific research, including the use of cobalt chloride to study the effects of hypoxia on respiratory plasticity and oxygen uptake in rat liver mitochondria [32], the study of cobalt chloride-induced hypoxic stress in rat myocardial H9c2 cells [33], and the study of the effect of the chemical inducer cobalt chloride and hypoxia on apoptosis in the human hepatocellular carcinoma cell line HepG2 [34]. Studies have pointed out that there is a relationship between FAM13A gene and HIF. FAM13A is involved in tumour cell promotion and downstream of TGFβand HIF1. In addition, we also used gepia online system to simulate the correlation between FAM13A and HIF, and found that the expression showed high correlation. Therefore, we used hypoxia inducer cobalt chloride to study the function of FAM13A [26]. In this study, the function of FAM13A in the proliferation of Qinchuan bovine precursor adipocytes was investigated. The study was performed using an overexpression plasmid vector and small interfering RNA. Then, the relevant signalling pathways were verified by transcriptome sequencing and other means. To verify the function of the signalling pathway in reverse, a cellular hypoxia model was constructed using cobalt chloride, a chemical inducer of hypoxia. This study clarified the function of FAM13A in the proliferation of precursor adipocytes in Qinchuan cattle and provided a theoretical basis for molecular breeding of beef cattle and improvement of beef adipose deposition and quality.

## 2. Materials and methods

### 2.1 Ethics statement

All animal handling was approved by Northwestern A&F University’s Experimental Animal Management Committee (EAMC). In accordance with the EAMC/121472 statement on 5 September 2019, all institutions and government regulations were followed.

### 2.2 Animals

Samples were collected from newborn Qinchuan calves at the breeding farm of the National Beef Improvement Center of Northwest Agriculture and Forestry University. Perirenal lipids were collected and precursor adipocytes were isolated according to routine laboratory methods, and stored in liquid nitrogen. Animal care and study protocols were approved by the Animal Care Commission of the College of Veterinary Medicine, Northwest Agriculture and Forestry University.

### 2.3 Primer design and antibody information

Primers were designed using Primer 5 (primer premier 5) and the National Center for Biotechnology Information primer tool (https://www.ncbi.nlm.nih.gov/tools/primer-blast/), including primers to amplify the complete coding sequence region of FAM13A for subsequent ligation into the overexpression vector. Primers related to cell proliferation, including minichromosome maintenance complex component 3 (MCM3), cyclin E1 (CCNE1), and cyclin dependent kinase 1 (CDK1) were designed and synthesized to study the effect of FAM13A on bovine precursor adipocyte proliferation. Ribosomal RNA 18s was selected as an internal reference control and a few differentially expressed genes were validated after RNA sequencing (RNA-seq). Thus, primers were designed for serpin family E member 1 (SERPINE1), pyruvate dehydrogenase E1 subunit beta (PDHB), enolase 3 (ENO3), insulin like growth factor 1 receptor (IGF1R), endothelin 1 (EDN1), and ENO2. Primers related to signalling pathways, such as HIF-1α and HIF-1β, were also included. Primer-related information is presented in Table 1.

**Table 1.** The primer sequence and information of FAM13A gene

Primer	Primer Sequence (5’-3’)	Annealing temperature (°C)
FAM13A-CDS-F	CTAGCGTTTAAACTTAAGCTTGCCAC CATGGCTTGTGAAATCATGCCT	56.2
FAM13A-CDS-R	AACGGGCCCTCTAGACTCGAG GATGGGGCTGACTCCTCACAT	58.4
18S-F	CCTGCGGCTTAATTTGACTC	57.1
18S-R	AACTAAGAACGGCCATGCAC	58.8
FAM13A-F	GTACCGCCTGGTCAAACAGATCCTA	64.1
FAM13A-R	TAGTTATCGTCTTCTGAACCCTC	57.1
CDK1-F	CAAAGCTGGCGCTTGGAAGTTAG	62.7
CDK1-R	GTAGACCCCGGCTTTATCCGC	62.9
MCM3-F	TGGTGACGCTATGCCTCTTG	60.1
MCM3-R	GGTCATCAGGGCTGAAGTTGG	60.9
CCNE1-F	CGATGTCTCTGTTCGCTCCA	55.0
CCNE1-R	CCACACTGGCTTCTCACAGT	55.0
SERPINE1-F	CACGCCTGGTCCTGGTAAATGC	61.7
SERPINE1-R	GGTGCTGCCATCGGACTTGTG	62.2
PDHB-F	AATTGCTGTAGGTGCCGCTATGG	60.2
PDHB-R	TTTATGACCTGGTCGATGGCTTGC	59.8
ENO3-F	GCTACCTGGACCTCGTTCCTCTC	61.8
ENO3-R	GACCTTCAGCAGCAGGCAGTTG	61.8
IGF1R-F	AACATCGCTTCGGAACTGGAGAAC	59.6
IGF1R-R	TCAGGAAGGACAAGGAGACCAAGG	60.8
EDN1-F	TGCTCCTGCTCTTCCCTGATGG	59.1
EDN1-R	CGGAACAACGTGCTCTGGAGTG	59.1
ENO2-F	CGCCTCTCCCTCCTTCCTCTTC	62.3
ENO2-R	ACCCAGACGCTCCACACAGAC	62.6
HIF-1α-F	GATTGCCGTCTGCTCTCCCT	61.9
HIF-1α-R	CGCCCTCCATGGTGAATCG	60.9
HIF-1β-F	TGTCATGTTCCGGTTTCGGT	59.9
HIF-1β-R	TAGCTGTGGGACCTAGCTGT	59.9

### 2.4 Plasmid construction and siRNA

FAM13A was overexpressed using the pcDNA3.1 vector. The pcDNA3.1-FAM13A recombinant vector was constructed using a homologous recombination kit (Cat: 121,416; TaKaRa, Dalian, China) and sequencing verification and agarose gel electrophoresis were performed. The overexpression efficiency was determined by qRT-PCR and Western blot. To study the function of FAM13A, the BLOCK-iT™ RNAi Designer website (http://rnaidesigner.thermofisher.com/rnaiexpress/) was used to design the small interfering RNA (siRNA), si-FAM13A; these sequences are listed in Table 2.

**Table 2.** si-FAM13A and control Sequence Information of FAM13A Gene

Primer	Primer sequence(5‘-3’)
si-sense	CCAGCUCACUCGAAGGAUU
si-antisense	AAUCCUUCGAGUGAGCUGG
NC-sense	UUCUCCGAACGUGUCACGU
NC-antisense	ACGUGACACGUUCGGAGAA

### 2.5 Cell culture and cell transfection

Bovine precursor adipocytes preserved in liquid nitrogen were subjected to cell recovery at 37°C plus 5% CO2 in medium comprising 90% Dulbecco’s modified Eagle medium /F12, 10% foetal bovine serum, and 1% penicillin. Transfection using Lipofectamine^TM^ 3000 (Cat: L3000015; Invitrogen; Carlsbad, CA) was performed when the cell density reached 70–80%, according to the manufacturer’s instructions. Cells were collected 24 h after transfection for subsequent experiments.

### 2.6 5-Ethynyl-2′-deoxyuridine (EdU) assay

Adipocyte proliferation was detected using the Cell-light EdU Apollo 567 In Vitro Imaging kit (Ribobio,Guangzhou, China), and the proportion of EdU-positive cells to the total number of cells was calculated using images taken by fluorescence microscopy. Significance was analysed using biological statistics. Independent sample t-test was used for significance analysis.

### 2.7 Quantitative real-time polymerase chain reaction (qRT-PCR) analysis

Total cellular RNA extraction was performed using the RNAiso Plus operating instructions (Cat: 9109; TaKaRa). The OD values and concentrations of the extracted RNA were tested. cDNA synthesis was performed using the PrimeScript RT reagent kit with gDNA Eraser (Cat: RR047Q; TaKaRa). qRT-PCR assays were performed using TB Green Premix Ex Taq II (Tli RNaseH Plus) (Cat: RR820A; TaKaRa), according to the manufacturer’s instructions. Quantification was performed using a CFX-96 Touch Real-Time PCR Detection System (Bio-Rad, Hercules, CA). Cq values obtained at the end of the experiment were calculated and analysed using the 2^−∆∆Cq^ algorithm. Finally, GraphPad Prism 6.0 was used for image generation.

### 2.8 Western blot analysis

The collected total proteins of adipocytes were boiled and inactivated, the protein samples in each lane were separated using 12% sodium dodecyl sulphate-polyacrylamide gel electrophoresis, and then transferred to a methanol-activated polyvinylidene fluoride membrane. The dilution ratio of the primary and secondary antibodies was according to the antibody production instructions. The antibody source information is presented in Supporting Information Table 3. Finally, luminescent liquid was added for exposure imaging and the imaging was statistically analysed using Image J software (GraphPad Prism 6.0) for image generation.

**Table 3.** Summary of antibody information

Antibody name	Purpose	Source
β-actin	primary antibody	Abcam
FAM13A	primary antibody	LSBio
CDK1	primary antibody	Abcam
CDK2	primary antibody	Abcam
PCNA	primary antibody	Abcam
Bad	primary antibody	Abcam
Bax	primary antibody	BBI
Bcl2	primary antibody	BBI
Bcl-XL	primary antibody	Abcam
HIF-1 alpha	primary antibody	Sigma
HIF-1 beta	primary antibody	Abcam
Goat Anti-Rabbit	secondary antibody	BBI
Goat Anti-Mouse	secondary antibody	Abcam

### 2.9 RNA-seq

Bovine preadipocytes were collected 24 h after transfection with si-FAM13A and sent to Gene Denovo Biotechnology Co (Gene Denovo, Guangzhou, China) for RNA-seq, differential expression, and pathway enrichment analysis. The sequencing platform selected was Illumina HiSeq2500 and the assembly accession was differentially expressed genes (DEGs). The selection false discovery rate (FDR) was < 0.05 and |Log2 fold change (log 2 FC)| > 0.5.

### 2.10 Screening of cobalt (II) chloride hexahydrate concentration

To investigate the mechanism of action of downstream signalling pathways after si-FAM13A transfection, cobalt (II) chloride hexahydrate was used to chemically induce hypoxia and create a cellular hypoxic environment. Cobalt (II) chloride hexahydrate (C8661; Sigma-Aldrich, shanghai, China) treatment was performed after the Qinchuan bovine precursor adipocyte density reached 70–80%. The concentration and duration of action of the added cobalt (II) chloride hexahydrate were determined and total RNA and total protein were collected from treated cells.

### 2.11 Gene ontology (GO) and KYOTO Encyclopaedia of Genes and Genomes (KEGG) pathway screening

Target genes identified from the RNA-seq data were subjected to GO enrichment and KEGG pathway analysis using the online Database for Annotation, Visualization and Integrated Discovery (https://david.ncifcrf.gov/) or the KEGG Orthology Based Annotation System 3.0 (http://kobas.cbi.pku.edu.cn/kobas3/?t=1). Screening was performed using P < 0.05. Finally, to identify the signalling pathways that function downstream after FAM13A interference and to analyse the correlation of FAM13A with key proteins of the HIF-1 signalling pathway, the public database analysis website Gene Expression Profiling Interactive Analysis (GEPIA) 2.0 (http://gepia2.cancer-pku.cn/#index) was used.

### 2.12 Statistical analysis

Experimental results are shown as the mean ± standard deviation (SD). Analysis of variance (ANOVA) and significance tests were performed using a two-tailed Student’s t-test or one-way ANOVA. Results were considered significant at P < 0.05 and very significant at P < 0.01. Analysis was performed using GraphPad Prism 6.0 (San Diego, CA) and SPSS 18.0 (IBM, California, USA).

## 3. Results

### 3.1 FAM13A promoted bovine preadipocyte proliferation

**Figure 1.**
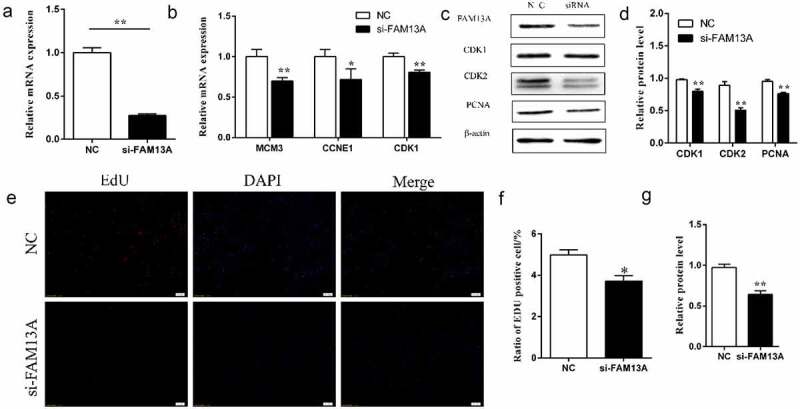
Family with sequence similarity 13 member A (FAM13A) interference inhibits bovine precursor adipocyte proliferation. (a) FAM13A interference efficiency, as measured after transfection with si-FAM13A. (b) mRNA levels of minichromosome maintenance complex component 3 (MCM3), cyclin E1 (CCNE1), and cyclin dependent kinase 1 (CDK1), which are genes related to cell proliferation, are detected after FAM13A interference. (c) protein levels of cell proliferation-associated proteins CDK1, CDK2, and proliferating cell nuclear antigen (PCNA) are detected by western blotting after FAM13A interference. (d) quantitative analysis of western blot results in panel C, as performed by Image J software. (e) the EdU kit stains cells in the proliferation phase. The red colour shows the EdU-labelled positive cells, the blue colour shows the 4′,6-diamidino-2-phenylindole (DAPI)-labelled nuclei, and merge is the combined superimposed result. (HD original picture see Figure S2) (f) quantitative analysis of EdU staining results using image J software. (g) western blot detecting the interference efficiency of the FAM13A protein. * indicates *P < 0.05* and ** indicates *P < 0.01.*

qRT-PCR results of si-FAM13A-transfected cells are shown in Figure 1(a); the siRNA interference efficiency was 83.5% (P < 0.01) allowing subsequent experiments to be performed. Total RNA and total protein were collected from cells after transfection with si-FAM13A or the control and levels of cell proliferation-related genes and proteins were examined; the results are shown in Figure 1(b-g). When FAM13A was knocked down, mRNA levels of cell proliferation-related genes, including MCM3 (P < 0.01), CCNE1 (P < 0.05), and CDK1 (P < 0.01) were significantly reduced. Western blotting showed that levels of CDK1, CDK2, and proliferating cell nuclear antigen (PCNA) were highly significant (P < 0.01).

(Figure 1(e)) & S2 demonstrate the EdU staining results, (Figure 1(f)) is the result of statistics of EdU stained positive cells using Image J software. It can be seen that the number of red positive cells is significantly lower than that of the control group after FAM13A interference (P < 0.05).

**Figure 2.**
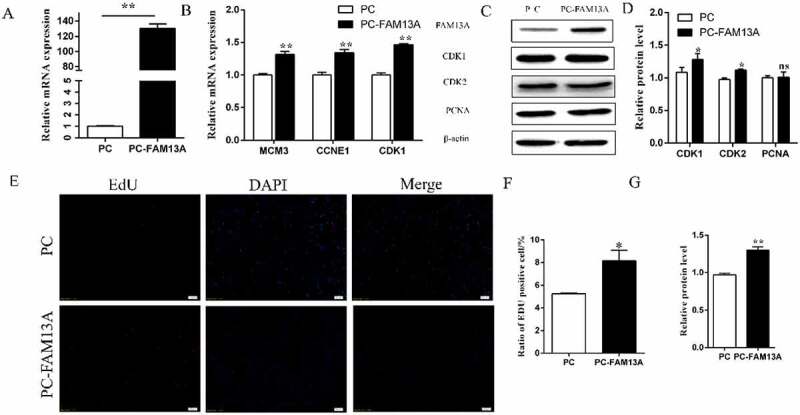
Family with sequence similarity 13 member A (FAM13A) overexpression using pcDNA3.1(+) promotes bovine precursor adipocyte proliferation. (a) FAM13A overexpression efficiency after transfection with FAM13A. (b) mRNA levels of the cell proliferation-related genes minichromosome maintenance complex component 3 (MCM3), cyclin E1 (CCNE1) and cyclin dependent kinase 1 (CDK1) are detected after FAM13A overexpression. (c) Protein levels of cell proliferation-associated proteins CDK1, CDK2, and proliferating cell nuclear antigen (PCNA) are detected by western blot after FAM13A overexpression. (d) quantitative analysis of the western blot results in panel C, as performed by Image J software. (e) the EdU kit stains the cells in proliferation phase. the red colour shows the EdU-labelled positive cells, the blue colour shows the 4′,6-diamidino-2-phenylindole (DAPI)-labelled nuclei, and merge is the combined superimposed result. (HD original picture see Figure S3) (f) Quantitative analysis of EdU staining results using image J software. (g) Western blot results to detect the FAM13A protein overexpression efficiency. * indicates *P < 0.05*, ** indicates *P < 0.01*, PC indicates pcDNA3.1 (+) empty vector, and PC-FAM13A indicates the recombinant vector

The pcDNA3.1-FAM13A recombinant overexpression vector and the control empty vector were also transfected into cells. The overexpression efficiency was 130.5-fold (P < 0.01), as shown in (Figure 2(a)), allowing subsequent experiments to be conducted. Total RNA and total protein were collected 24 h after transfection; the results are shown in (Figure 2(b-g)). Using qRT-PCR, the proliferation-related genes MCM3, carotenoid cleavage dioxygenase 1 (CCDE1), and CDK1 were significantly elevated (P < 0.01), while at the protein level changes in CDK1, CDK2, and PCNA levels were detected. CDK1 and CDK2 were significantly elevated in the overexpression group (P < 0.01), but PCNA levels were not significantly elevated. EdU staining results are shown in (Figure 2(e)) & S3 and (Figure 2(f)) are the positive cells stained by EdU by Image J software. Consistently, levels of EdU-stained positive cells were significantly higher than those in the control group (P < 0.05). The above results indicated that interfering with FAM13A expression inhibited Qinchuan bovine precursor adipocyte proliferation while FAM13A overexpression by the pcDNA3.1 vector promoted the proliferation of Qinchuan bovine precursor adipocytes.

### 3.2 FAM13A promoted proliferation through the HIF-1 signalling pathway

**Figure 3.**
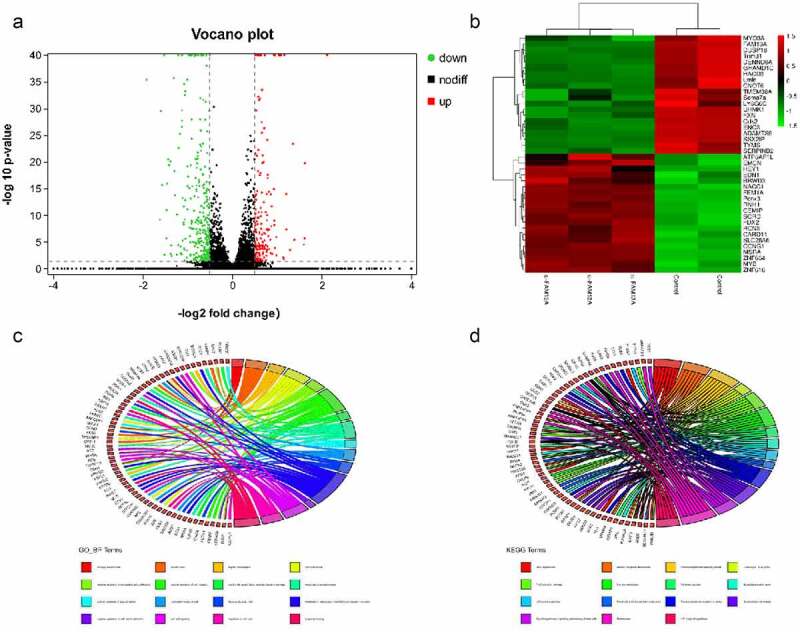
RNA sequencing results after family with sequence similarity 13 member A (FAM13A) interference. (a) volcano plot of differential genes after RNA sequencing, where green indicates downregulation, red indicates upregulation, and black indicates an insignificant difference. (b) heat map indicating upregulation and downregulation of the Top 20 differential genes. darker green indicates lower expression in the group, and darker red indicates higher expression in the group. (c) gene ontology (GO) enrichment analysis of differentially expressed genes and string plot presentation using R. (d) Kyoto Encyclopaedia of Genes and Genomes (KEGG) pathway enrichment analysis performed for some differentially expressed genes and is presented as a string plot using R

**Figure 4.**
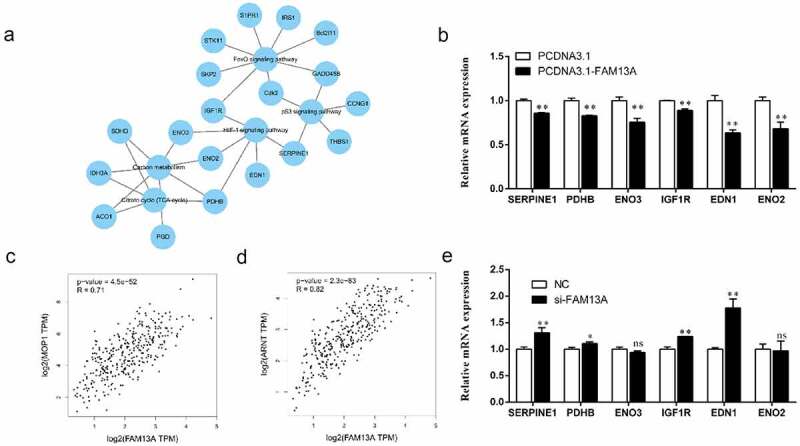
RNA sequencing results of cells with family with sequence similarity 13 member A (FAM13A) interference are validated against fluorescent quantitative polymerase chain reaction (PCR) results. (a) the main pathways enriched for differentially expressed genes, including hypoxia inducible factor-1 (HIF1), tricarboxylic acid cycle, carbon metabolism, Forkhead box O (FoxO), and p53 signalling. (b, e) fluorescence quantitative PCR validation of the differentially expressed genes enriched in the HIF-1 signalling pathway. (c) scatter plot of the correlation between HIF-1α (MOP1), a key gene of the HIF-1 signalling pathway, and FAM13A. (d) scatter plot of the correlation between HIF-1β (ARNT), a key gene of the HIF-1 signalling pathway, and FAM13A. * indicates *P < 0.05* and ** indicates *P < 0.01.*

To investigate the inhibition of precursor adipocyte proliferation through a mechanism or pathway after FAM13A interference, transcriptome sequencing of the samples after transfection with si-FAM13A was performed. Principal component analysis between samples suggested that there was relatively poor repetition in the control group so it was discarded in subsequent analyses. According to the screening conditions, 491 DEGs are identified, among which, 171 are upregulated and 320 are downregulated, as shown in Figure 3. Figure 3(a) shows the volcano plot of these genes, in which red dots indicate differentially upregulated genes, green dots indicate differentially downregulated genes, and the black part in the middle indicates genes with insignificant differences. Figure 3(b) shows a heat map of the Top 20 upregulated and downregulated DEGs; the upregulated genes expressed in the si-FAM13A group are Hes related family BHLH transcription factor with YRPW motif 1, EDN1, nucleus accumbens associated 1, sorbitol dehydrogenase, and reticulocalbin 3. The downregulated genes were myosin IIIA, tripartite motif containing 31, 3-hydroxyacyl-CoA dehydratase 3, CCR4-not transcription complex subunit 6, and CDK2.

(Figure 3(c)) shows the GO and KEGG enrichment analysis of DEGs and the string diagram created using the R. GO terms were mainly enriched in biological processes related to cell cycle regulation, as well as regulation of RNA polymerase II promoter transcription. (Figure 3(d)) shows the results of KEGG pathway enrichment analysis, demonstrating the enrichment of RNA degradation, tricarboxylic acid (TCA) cycle process, p53, Forkhead box O (FoxO), and HIF-1 signalling pathways. A protein–protein interaction network was constructed on the DEGs enriched in the following pathways, including the TCA cycle, HIF-1 signalling, carbon metabolism, and FoxO signalling; the results are shown in (Figure 4(a)). To further verify the transcriptome sequencing data, qPCR was performed to verify mRNA levels of genes enriched in the HIF-1 signalling pathway; the qPCR results after FAM13A interference are shown in (Figure 4(e)). Compared to that of the control group, SERPINE1, IGF1R, and EDN1 were highly significantly elevated (P < 0.01), PDHB was significantly elevated (P < 0.05), while ENO3 and ENO2 were not significantly elevated. The mRNA qPCR results of FAM13A overexpression are shown in Figure 4(b); all of the above genes are significantly decreased (P < 0.01).

To further identify key signalling pathways, the correlation of FAM13A with HIF-1α (MOP1) and HIF-1β (ARNT), key proteins in the HIF-1 signalling pathway, were analysed using the human cancer online database GEPIA 2.0 and the Spearman’s correlation coefficient. Whole blood samples have been selected for analysis and the results are shown in (Figures 4(c-d)). The correlation R-value of FAM13A with HIF-1α (MOP1) was 0.71, P = 4.5 × 10^−52^. The correlation R-value of FAM13A with HIF-1β (ARNT) was 0.82, P = 2.3 × 10^−83^. Therefore, the HIF-1 signalling pathway was selected for validation.

**Figure 5.**
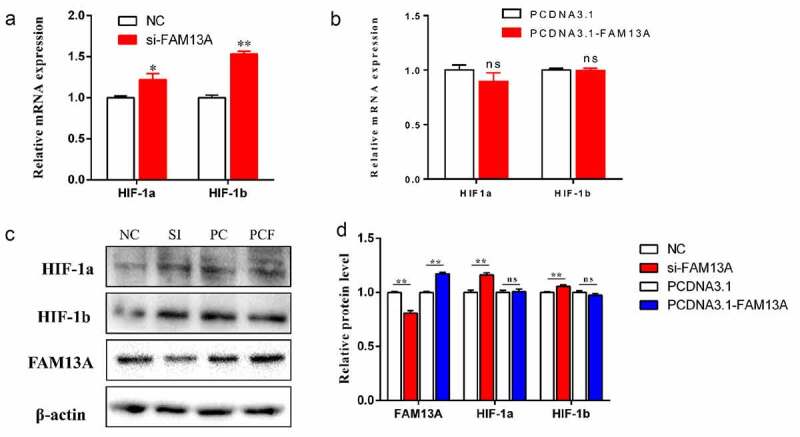
Family with sequence similarity 13 member A (FAM13A) interference promotes cellular hypoxia. (a) changes in mRNA levels of hypoxia-associated genes hypoxia inducible factor-1 (HIF1α) and HIF1β are detected after FAM13A interference. (b) changes in mRNA levels of hypoxia-associated genes HIF1α and HIF1β are detected after FAM13A overexpression. (c) changes in hypoxia-related proteins HIF-1α and HIF-1β, as well as the FAM13A protein are detected using western blotting after FAM13A interference and overexpression. The internal reference protein is β-actin.(NC: normal contrast, SI: si-FAM13A, PC: PCDNA3.1, PCF: PCDNA3.1-FAM13A) (d) quantitative analysis of the results in Figure c using image J software. * indicates *P < 0.05* and ** indicates *P < 0.01.*

Bovine precursor adipocytes were also transfected at a density of 70% with si-FAM13A and pcDNA3.1-FAM13A recombinant vectors and the control vectors, after which, the mRNA and protein levels of HIF-1α and HIF-1β were examined. The results are shown in (Figure 5(a-d)). The expression of HIF-1α (P < 0.05) and HIF-1β (P < 0.01) in the si-FAM13A group was significantly increased and the overexpression group was not significantly different; similar results were observed at the protein level. The above results indicated that FAM13A interference increased the HIF-1 signalling pathway by increasing the expression of the key protein, HIF-1β, leading to slower cell proliferation.

### 3.3 Establishment of a bovine primary adipocyte anoxia model

**Figure 6.**
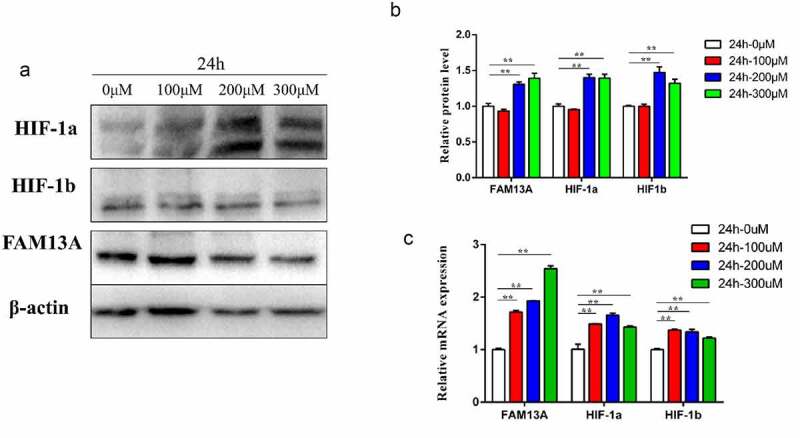
Addition of cobalt chloride negatively regulates and thus, increases family with sequence similarity 13 member A (FAM13A) protein expression. (a) after the addition of 0 μM, 100 μM, 200 μM, and 300 μM cobalt chloride, the proteins are collected at 24 h, respectively, and the changes in FAM13A protein and hypoxia-inducible related proteins hypoxia inducible factor-1 (HIF1α) and HIF-1β are detected; *β-actin* is selected as the internal reference protein. (b) quantitative analysis of western blot results at 24 h after the addition of cobalt chloride using image J software. (c) changes in FAM13A mRNA levels and the hypoxia-associated genes HIF1α and HIF1β are detected after 24 h addition of cobalt chloride. * indicates *P < 0.05* and ** indicates *P < 0.01.*

To further verify whether FAM13A affected bovine precursor adipocyte proliferation through the HIF-1 signalling pathway because the HIF-1 signalling pathway was a hypoxia-related signalling pathway, the hypoxia-mimicking chemical inducer cobalt chloride was used to treat cultured bovine precursor adipocytes. First, to determine the optimal concentration and duration of action, a concentration gradient was performed. The concentration gradient included: 0 μM, 100 μM, 200 μM, 300 μM and the action time was 24 h. The bright field test was performed for cell viability, and the results are shown in Figure S1. Cells were damaged and most cells were dead at concentrations greater than 400 μM. Therefore, 0 μM, 100 μM, 200 μM, and 300 μM concentrations were selected for total RNA and total protein extraction. Figure 6 shows the samples collected after 24 h of cobalt chloride treatment. Using qRT-PCR, compared to that of the 0 μM control group, there was a significant increase at 100, 200, and 300 μM concentrations (P < 0.01), and the protein levels were highly significant, except at 100 μM, which was not significantly different.

### 3.4 FAM13A upregulation in bovine primary adipocytes after hypoxia

Previously, the appropriate concentration and duration of cobalt chloride treatment was determined to induce a hypoxic state in cells. To further determine whether there was an effect on FAM13A expression when cells were in a hypoxic state, FAM13A expression when exposed to different concentrations of cobalt chloride treatment was simultaneously examined. As shown in (Figure 6(a-c)), FAM13A mRNA levels were significantly increased by 200 μM and 300 μM at 24 h. A concentration of 100 μM cobalt chloride did not affect FAM13A protein levels. From the above experimental results, it was concluded that when cells were exposed to hypoxia, FAM13A mRNA and protein levels were increased in cells.

## 4. Discussion

Studies on FAM13A have mainly focused on COPD [15,16]. In recent years, studies have reported that this gene is closely related to metabolism [35]. However, studies in cell proliferation are rarely reported.

Here, the role of FAM13A in the proliferation of bovine precursor adipocytes was investigated by designing and synthesizing FAM13A siRNA, as well as the pcDNA3.1 overexpression recombinant vector. After FAM13A interference, precursor adipocyte proliferation was inhibited, as determined by EdU assay, fluorescent qRT-PCR, and western blotting. In contrast, precursor adipocyte proliferation was promoted in bovine precursor adipocytes transfected with pcDNA3.1 recombinant plasmids overexpressing FAM13A. The HIF-1Α signalling pathway was identified using transcriptome sequencing of samples from si-FAM13A-transfected cells and GO and KEGG enrichment analysis. The correlation of FAM13A with HIF-1α and HIF-1β, key proteins of the HIF-1Α signalling pathway, was analysed using the GEPIA 2.0 database, and changes in mRNA and protein levels of these key proteins after transfection with si-FAM13A were verified using fluorescence qRT-PCR and western blotting. The expression of both HIF-1α and HIF-1β was significantly increased, whereas the overexpression group was not significantly different; similar results were observed at the protein level. These data suggested that FAM13A interference led to slower cell proliferation by increasing the expression of HIF-1β, a key protein in the HIF-1 signalling pathway. Since HIF-1α belongs to the hypoxic signalling pathway, the chemical mimetic cobalt chloride was used to construct a cellular hypoxia model to determine whether the hypoxic process had a regulatory effect on FAM13A expression. Samples were collected to detect the mRNA and protein levels of FAM13A. When cells were exposed to hypoxia, there was an increase in the mRNA and protein levels of FAM13A in these cells. The study of FAM13A in cell proliferation was reported in 2016. Inhibition of FAM13A in NSCLC inhibits tumour cell proliferation, FAM13A levels are directly associated with HIF-1α levels, and FAM13A is located downstream of HIF-1α [26]. Another study has revealed that FAM13A is involved in COPD remodelling by affecting the proliferation of human airway epithelial cells [25]. Studies on FAM13A regulatory mechanisms have moved from human COPD [16], fibrosis of the lung [36], and NSCLC [26,37], to insulin sensitivity [38], adipocyte differentiation [19,23], and proliferation [39,40], and the correlation between the hypoxic state and other aspects [35,37,41]. In this study, precursor adipocytes from bovine animal models were used as test materials for cell proliferation and hypoxia-related studies. For functional studies, overexpression using recombinant plasmids had a general effect, but some plasmids were not successful, so subsequently, viruses were constructed for validation.

During transcriptome data processing and screening, the HIF-1 signalling pathway was not identified since the P > 0.05. Thus, the literature and the GEPIA 2.0 database [38,40]. were used to analyse FAM13A and the HIF-1 signalling pathway key proteins HIF-1α (MOP1) and HIF-1β (ARNT), which identified the possible involvement of FAM13A and the HIF-1 signalling pathway in precursor adipocyte proliferation.

In a study investigating the protective effect of arjunic acid on cobalt chloride-induced hypoxic injury and apoptosis in rat cardiomyocytes, treatment of cells with 1.2 mM cobalt chloride for 24 h induced cytotoxicity [33]. In another study using ursodeoxycholic acid to protect cardiomyocytes from cobalt chloride-induced hypoxia, treatment with cobalt chloride for 24 h also induced cytotoxicity [42]. In this study, mRNA and protein levels of HIF-1α and HIF-1β were not detected in bovine precursor adipocytes at significant levels after 12 h of cobalt chloride treatment. It is possible that the transfection time was not long enough to replace Fe^2+^ with Co^2+^ in PHDs to cause cellular hypoxia.

**Figure 7.**
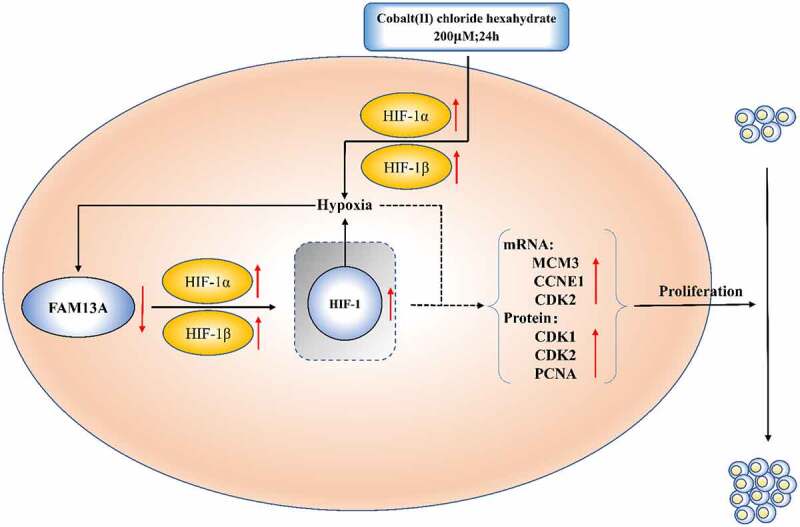
Pathway diagram of family with sequence similarity 13 member A (FAM13A) promoting bovine preadipocyte proliferation through the hypoxia inducible factor-1 (HIF-1) signalling pathway

In this study, a bovine precursor adipocyte hypoxia model was constructed using the chemical inducer cobalt chloride and FAM13A mRNA and protein levels were elevated to different degrees when the cells were exposed to hypoxia. The elevated FAM13A mRNA levels inhibited hypoxia through negative feedback of HIF-1β to alleviate cellular hypoxia, and the elevated FAM13A mRNA and protein levels promoted the proliferation of precursor adipocytes. This FAM13A regulatory process is shown in Figure 7.

This study systematically complemented the function of FAM13A in cell proliferation and hypoxia and lays a theoretical foundation for subsequent studies.
